# Plant disease prescription recommendation based on electronic medical records and sentence embedding retrieval

**DOI:** 10.1186/s13007-023-01070-6

**Published:** 2023-08-26

**Authors:** Junqi Ding, Yan Qiao, Lingxian Zhang

**Affiliations:** 1 China Agricultural University, Beijing, 100083 China; 2 Beijing Plant Protection Station, Beijing, 100029 China; 3 Key Laboratory of Agricultural Informationization Standardization, Ministry of Agriculture and Rural Affairs, Beijing, China; 4 College of Information and Electrical Engineering, China Agricultural University, 209# No.17 Qinghua Donglu, Haidian District, Beijing, 100083 China

**Keywords:** Plant disease, Prescription recommendation, Sentence embedding, CoSENT

## Abstract

**Background:**

In the era of Agri 4.0 and the popularity of Plantwise systems, the availability of Plant Electronic Medical Records has provided opportunities to extract valuable disease information and treatment knowledge. However, developing an effective prescription recommendation method based on these records presents unique challenges, such as inadequate labeling data, lack of structural and linguistic specifications, incorporation of new prescriptions, and consideration of multiple factors in practical situations.

**Results:**

This study proposes a plant disease prescription recommendation method called PRSER, which is based on sentence embedding retrieval. The semantic matching model is created using a pre-trained language model and a sentence embedding method with contrast learning ideas, and the constructed prescription reference database is retrieved for optimal prescription recommendations. A multi-vegetable disease dataset and a multi-fruit disease dataset are constructed to compare three pre-trained language models, four pooling types, and two loss functions. The PRSER model achieves the best semantic matching performance by combining MacBERT, CoSENT, and CLS pooling, resulting in a Pearson coefficient of 86.34% and a Spearman coefficient of 77.67%. The prescription recommendation capability of the model is also verified. PRSER performs well in closed-set testing with Top-1/Top-3/Top-5 accuracy of 88.20%/96.07%/97.70%; and slightly worse in open-set testing with Top-1/Top-3/Top-5 accuracy of 82.04%/91.50%/94.90%. Finally, a plant disease prescription recommendation system for mobile terminals is constructed and its generalization ability with incomplete inputs is verified. When only symptom information is available without environment and plant information, our model shows slightly lower accuracy with Top-1/Top-3/Top-5 accuracy of 75.24%/88.35%/91.99% in closed-set testing and Top-1/Top-3/Top-5 accuracy of 75.08%/87.54%/89.84% in open-set testing.

**Conclusions:**

The experiments validate the effectiveness and generalization ability of the proposed approach for recommending plant disease prescriptions. This research has significant potential to facilitate the implementation of artificial intelligence in plant disease treatment, addressing the needs of farmers and advancing scientific plant disease management.

## Background

Plant disease management is critical to meeting the challenges of sustainable agriculture, and one of the keys is a science-based disease control and treatment strategy. The selection of appropriate treatment plan for plant diseases in various environments and with different symptoms relies on specialized disease knowledge. However, our past research has shown that many producers choose pesticide application strategies based on personal experience, advice from friends and advertising, lacking a reliable source of knowledge and information [1]. Consequently, the scope and norms of pesticide use are ignored, which may increase the risk of contaminating the environment and endangering human health [2]. The diffusion of science- and evidence-based plant production management practices is challenging especially in areas dominated by smallholder farming [3]. Accurate recommendation of pesticide prescriptions is an important issue that needs to be addressed in agricultural management.

To assist smallholder farmers in managing plant diseases, Plantwise^1^, a global program led by the Center for Agriculture and Biosciences International (CABI), has established plant clinics in over 30 countries in Africa, Asia and Latin America, creating a global network of plant clinics operated by professional plant doctors [4, 5]. These plant doctors play a crucial role in providing farmers with recommendations for effective pest and disease management. They prescribe targeted pesticide application strategies, called prescriptions, tailored to the specific circumstances and symptoms of disease events. This valuable information, concerning plant diseases and their management, is organized and recorded as Plant Electronic Medical Records (PEMR), which usually consist of the following key sections:

1. Disease Symptoms: PEMR includes descriptions of disease symptoms observed in plants, such as leaf spots, wilting, rotting, and slow growth. These symptoms serve as the primary and direct basis for disease diagnosis and subsequent treatment strategies and are also the main objects of most intelligent disease diagnosis research [6, 7].
2. Environmental characteristics: Environmental factors, including the season and geographic location of affected plants, can influence disease development and aid in determining appropriate management strategies [8].
3. Crop cultivation descriptions: PEMR contains details about the cultivation practices employed, including whether the crops are grown in an open field or a greenhouse. Different cultivation methods may require distinct management approaches [9].
4. Diagnosed Diseases: The specific diseases diagnosed by plant doctors, such as cucumber powdery mildew or tomato blight, are documented in PEMR. This information facilitates tracking the occurrence and prevalence of different diseases over time.
5. Recommended Pesticides: Includes both biological and chemical pesticides, which may be selected by the plant doctor depending on the severity of the disease.
6. Application Methods: PEMR provides instructions on how to apply the recommended pesticides. This information covers details such as the frequency of application (e.g., once every 5–7 days), the duration of the treatment (e.g., consecutive application for 3 times), and any specific application techniques or precautions.
7. Field management measures: PEMR may also incorporate field management measures crucial for disease prevention and control, such as greenhouse isolation, ventilation, and cooling.

The above key elements in PEMR provide a valuable source of information for intelligent plant disease management research [10]. By analyzing and mining the historical data of PEMR, it is possible to summarize the patterns of disease medication for similar diseases, plants and environments, and then realize intelligent prescription recommendations. How to effectively mine the PEMR data is an urgent problem to be solved.

Currently, data mining research in electronic medical records (EMRs) focuses on the human healthcare domain [11–13], especially on various prescription recommendation tasks such as recommendations for diabetes prescriptions [14], TCM prescriptions [15–17] and Parkinson's disease prescriptions [18], drugs for cancer cell lines [19]. Zhao et al. predicted herbal prescriptions in the form of probability values by graph convolution construction and multilayer perceptron (MLP) [15]; He et al. proposed a machine learning method called kernelized ranking learning (KRL) to formulate personalized drug recommendations as a ranking problem [19]; Ye et al. combined knowledge graph (KG) and recommender system for drug-target interaction prediction [20]; Shi et al. recommended the prescription of typical drugs by learning the relationship between observed symptoms and prescribed drugs through multimodal representation [18]. Most of these existing methods for EMR make good progress by extracting features from typical symptom representations and then classifying, ranking or predicting the prescriptions. However, the specificity of PEMRs makes plant disease prescription recommendation a challenging task, and existing methods face four main issues:

1. Lack of labeling data. Unlike human medical institutions, electronic information systems are not widely available in plant disease control institutions, resulting in a lack of publicly available PEMR datasets. Current intelligent prescription recommendation models usually rely on a large amount of labeled training data, which is not feasible for plant disease prescription recommendation tasks where labeled data are lacking.
2. Lack of structural and linguistic specifications. In contrast to EMR in the medical domain, PEMR lacks strict structural specifications. In addition, PEMR texts often contain a large number of dialects and slang from agricultural production, which makes it a challenge to extract semantic features by combining agricultural knowledge.
3. Challenge of adding new prescriptions. Current intelligent prescription recommendations have fixed prescription categories that cannot easily accommodate new prescriptions. Adding new prescriptions requires a large amount of label data for the new prescriptions and requires the network to be modified and retrained. However, in production practice, pesticide categories are diverse and frequently change, and label data for many new pesticides are difficult to obtain. These problems make it challenging for existing prescription prediction models to achieve flexible adjustments of pesticide prescription categories and quantities.
4. Multiple factors need be considered in practical application scenarios. The disease triangle principle in plant pathology states that the manifestation of plant diseases is affected by a combination of host genetic susceptibility, pathogen virulence, and abiotic environmental parameters. It is important to note that the same pathogen may necessitate different treatment approaches depending on various factors, such as the crop type, season, temperature, and field distribution. Consequently, it is crucial to conduct comprehensive comparisons and tests when making prescription recommendations in practical settings.

To address these challenges, we conducted a thorough survey of relevant research in the field of plant disease diagnosis and prescription recommendation. The majority of research on intelligent plant disease diagnosis centers around digital image processing [6, 7, 21], while only a few studies explore mining PEMRs. For example, Xu et al. [10] applied a two-phase stacked integrated learning approach to mine structured prescription data related to tomato diseases. Additionally, Ding et al. [22] proposed a crop disease diagnostic model, CdsBERT-RCNN, for mining the text information of crop EMRs, thereby establishing a foundation for feature extraction from PEMRs.

Regarding prescription recommendations, several typical approaches have been explored. Rule-based and knowledge graph-based recommendation methods require substantial manual effort, face scalability issues, and are not suitable for PEMRs lacking structural and linguistic specifications [23–28]. On the other hand, instance-based recommendation methods are simple and effective [13, 29], and with the introduction of deep learning and pre-trained language models, they can extract deep semantic features [22, 30, 31], thereby addressing the lack of labeled PEMR data. Some studies have transformed the prescription recommendation problem into an EMR classification task [16, 32, 33], but this approach can only recommend trained prescription categories. In contrast, semantic matching-based recommender systems offer more flexibility in adding new prescriptions [34, 35].

Semantic matching methods can be categorized as representation-based [36, 37] and interaction-based approaches [38, 39]. The former generates sentence embeddings by pre-processing the dataset to reduce online computation time, making it suitable for large-scale retrieval. The latter incurs higher computational costs and is suitable for small-scale retrieval or ranking. We attempted an interaction-based matching method, but experimental results indicated that, given limited computational resources, the online computation time required to complete our prescription recommendation task would take several years. Further details on the related work are provided in the following section.

In summary, there is a lack of suitable intelligent methods for pesticide prescription recommendation in plant disease management. Therefore, this paper aims to develop a plant disease prescription recommendation method based on sentence embedding retrieval (PRSER) to provide accurate and personalized recommendations for pesticide application strategies, enabling smallholder farmers to make informed decisions regarding disease treatment. To leverage the information captured in PEMRs, we choose a representation-based semantic matching approach to improve the speed of prescription retrieval. Also, a combination of a pre-trained language model (PLM) and a contrast learning approach is used to achieve effective sentence embedding in PEMRs. By addressing these challenges, we expect to make significant contributions to intelligent disease management in the agricultural domain.

## Related work

### Prescription recommendations

Current prescription recommendation methods can be classified as rule-based, knowledge graph-based and instance-based recommendations. Rule-based recommendation methods, known as expert systems, are widely used in research on plant disease treatment recommendation, such as apple diseases [23], pulse plant diseases [40] and oilseed-plant diseases [41]. Expert systems utilize pre-determined rules for matching, requiring a great deal of manual effort to ensure the construction and knowledge updating of the expert pool [23], and are limited by rules when applied [24].

Knowledge graphs cover a more comprehensive range of knowledge than traditional expert systems. To support the construction of knowledge graphs, existing studies have identified entities [42–44] and relationships [45–47] between drugs and diseases from various data sources. In downstream tasks, knowledge graphs can be combined with algorithms such as machine learning to achieve prescription recommendations [25–27]. At the same time, knowledge graph-based recommendations face challenges such as high computational complexity, lack of long-tail entities, rule conflicts, difficulty in extension and limitations of application in unstructured EMR [28].

The example-based recommendation approach introduces machine learning to achieve intelligent recommendations based on drug prescriptions given in existing EMRs. [29] utilized similarity algorithms to predict disease-drug interactions; [48] constructed a user-adaptive medication recommendation systems based on inference from Bayesian networks. Ref. [13] proposed an extended treatment recommendation model based on reinforcement learning using electronic health records from the South Korean health insurance system. With the development of natural language processing techniques, deep learning and pre-trained language embeddings have been applied to solve complex natural language processing (NLP) tasks, including in the fields of plant disease management [22, 49–51] and drug recommendation [28, 30, 31, 52]. In particular, semantic matching has been proven to be efficient in a variety of recommendation systems [30, 34, 35].

### Semantic matching

As one of the fundamental problems in the field of NLP, semantic matching is widely used in downstream tasks such as information retrieval, recommender systems and question and answer systems [37, 53–55].

In general, there are two types of semantic matching models: representation-based and interaction-based. Representation-based models emphasizes the construction of the representation layer, encoding the text into overall embedding tensors before matching them, led by Microsoft's DSSM [36]. A series of models such as CDSSM [56], LDR-LTM [57] and Enhanced-DSSM [37] have since emerged, which have similar structures to DSSM, but replace the expression or matching layer with a more complex and effective algorithm. In retrieval and recommendation tasks, representation-based models can pre-process text with trained sentence embedding models to build indexes and significantly reduce online computation time.

Interaction-based models interact two texts at different granularities through a structure represented by an attention mechanism, aggregated into feature matrices that enters the representation layer to obtain the final relevance evaluation, e.g. ARC-II [38], ESIM [39], BiMPM [58]. Interactive computing better captures the semantic focus, but with high computational costs.

### Sentence embedding with PLMs

Bidirectional Encoder Representations from Transformers (BERT) [59], introduced by Google in 2018, brings research in natural language processing into the era of pre-trained language models (PLMs). Pre-training models by resorting to large-scale corpora in general-purpose domains and then fine-tuning them for downstream tasks has become the dominant paradigm in NLP [60]. An enormous amount of research effort has gone into it; examples include Robustly optimized BERT pre-training Approach (RoBERTa) [61], text-to-text transfer transformers (T5) [62], Knowledge-Enabled BERT (K-BERT) [63], clinical BERT embeddings (ClinicalBERT) [64].

Some studies have applied BERT to text matching, an example is the BERT-based interactive medical text-matching model constructed by [65], in which two sentences are joined by [sep] to form a sentence pair as model input. Such approaches require significant computational resources and struggle to make real-time inferences, which can be alleviated by the representation-based twin-tower structure with sentence embedding at its core [66]. Sentence-BERT [67] obtains sentence embedding by siamese and triplet network structure and the semantic similarity of two sentences can be gauged by the cosine similarity between their embeddings [68]. A Simple contrastive learning framework of sentence embedding (SimCSE), including both unsupervised and supervised versions, was proposed by Gao et al. in [69], achieving the SOTA performance of sentence-level semantic representation based on contrast learning and dropout data augment [70].

## Materials and methods

### Data processing and dataset construction

#### Data sources

The samples of plant electronic disease records in this paper come from 115 plant clinics established by Beijing Plant Protection Station. The plant clinics adopt a public welfare plant disease and pest diagnosis and consultation service model, in which qualified plant doctors to provide technical services for pest and disease control to farmers [4, 5]. The plant doctors make the diagnosis and finally prescribe the plant disease based on his personal experience, expertise, and references such as the Disease Understanding Paper prepared by the CABI Plantwise website.

This paper collects data for approximately 44 months between November 2017 and July 2021, resulting in a total of more than 44,000 PEMRs. PEMR records the pest and disease problems encountered by farmers in the process of plant cultivation and the treatment advice provided by plant doctors in accordance with specifications, including the main symptoms, geographical location, onset date, plant species, growth stage, affected parts, disease scale (mu), severity, field distribution, diagnosis results, consultation records, prescription of pesticides, pesticide quantity and other relevant information.

#### Data cleaning

The data were manually entered by numerous plant doctors and had various issues such as missing, redundant, and incorrect characters. We eliminate some records and fields with an excessive missing rate, de-duplicate the redundant records, and normalize the text. Furthermore, we standardize the pesticide names and eliminated invalid data to ensure the quality of the dataset, particularly for instances where prescription names are inaccurate, plant information is abnormal, and other problems existed.

#### Dataset construction

**Fig. 1:**
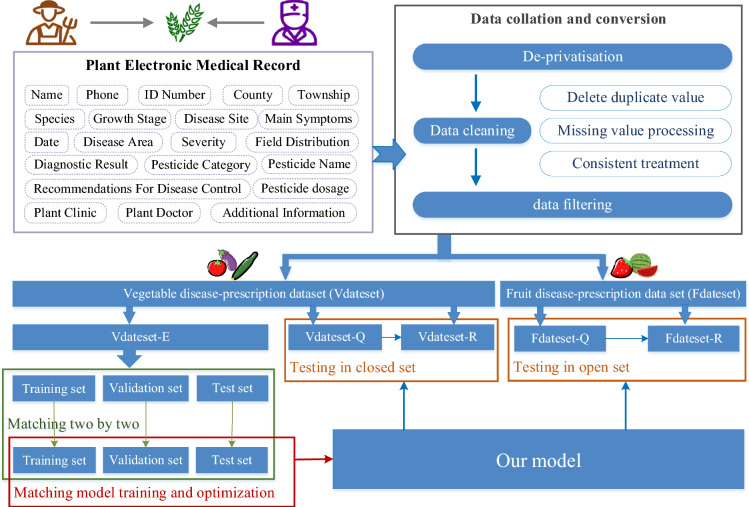
Data processing and data set partitioning

Two PEMR datasets are constructed as shown in Fig. 1: a vegetable disease-prescription dataset including tomato, cucumber, and eggplant with 20,791 instances, and a fruit disease-prescription dataset containing strawberry and watermelon with 1548 instances. The vegetable disease-prescription dataset (Vdateset) is divided into an experimental set (Vdateset-E), a query set (Vdateset-Q) and a reference set (Vdateset-R) in the ratio of 8:1:1. The fruit disease-prescription dataset (Fdateset) is divided into a query set (Fdateset-Q) and a reference set (Fdateset- R) in the ratio of 5:5.

The datasets are specifically used in the following scenarios:

1. Experimental dataset

Vdateset-E is used to train the semantic matching model based on sentence embedding. Stratified sampling is used to divide Vdateset-E into a training set (Vdateset-E-training) and a test set (Vdateset-E-test) with the ratio of 7:3. The training set data are paired with the text within the dataset by two-by-two matching. Text pairs corresponding to the same prescription are labeled as 1 and those corresponding to different prescriptions are labeled as 0. The ratio of positive and negative samples is adjusted to achieve balance. The test set is processed similarly. Finally, 173,708 text pairs for the training set and 74,281 text pairs for the test set are obtained for the semantic matching model.

(2) Closed-set testing.

Vdateset-Q and Vdateset-R are used for closed-set testing, with the former is the query set (i.e., the test set for prescription recommendation) and the latter as the reference set. The algorithm's ability to recommend prescriptions in closed sets is verified by analyzing experiments on Vdateset-E and Vdateset-Q, which belong to the same class.

(3) Open-set testing.

Fdateset-Q and Fdateset-R are used for open-set testing. Since diseases and prescriptions from the fruit disease prescription dataset do not appear in Vdateset-E, the generalization ability of our prescription recommendation system can be well evaluated.

### Prescription recommendation process

**Fig. 2:**
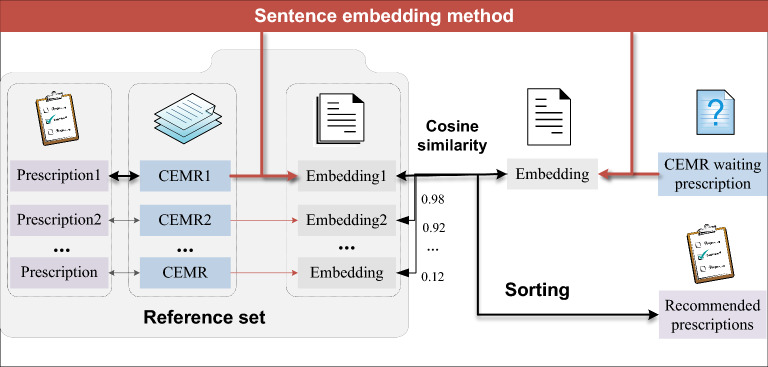
Prescription recommendation process

As shown in Fig. 2, our prescription recommendation system consists of three main components: semantic matching network training, construction and embedding of the reference set, and prescription recommendation for application scenarios. Specifically, the process is as follows:

1. Training PEMR sentence embedding method based on Vdateset-E.
2. Construct a standard reference set and vectorize all samples in the reference set using the trained sentence embedding scheme. The construction of the reference set is described in detail in 3.1.3.
3. Vectorize PEMR waiting prescription using the trained sentence embedding scheme, calculate their cosine similarity with all the reference vectors, and recommend the prescription corresponding to the highest similarity.

### PEMR sentence embedding method

To effectively train the PEMR sentence embedding method, we adopt a contrastive learning approach. The utilization of contrastive learning is motivated by its ability to learn powerful representations by contrasting positive and negative examples. By encouraging similar sentences to be closer to each other in the embedding space while pushing dissimilar sentences apart, the proposed method aims to capture the underlying semantic meaning of PEMR sentences.

**Fig. 3:**
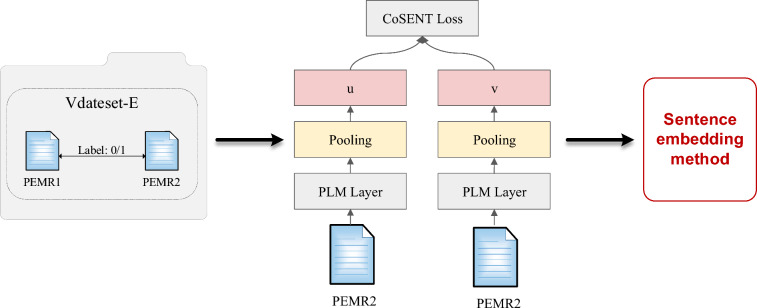
PEMR sentence embedding method

As depicted in Fig. 3, several essential components are included in our proposed approach to create a comprehensive and effective sentence embedding model. These components include a PLM (Pre-trained Language Model) Layer, a pooling operation, and the CoSENT loss function. The model structure and training process are described in detail in Sect. "PLM layer"-"Training process".

#### PLM layer

MacBERT (MLM as correction BERT) is a PLM adapted to Chinese, which maps text to vector space and converts each word into a vector of fixed dimension. MacBERT shares the same model structure as BERT, but modified two pre-training tasks in BERT: masked language model (MLM) and next sentence prediction (NSP).

1. MLM in BERT randomly masks 15% of the words in the input sequence, and then learns to predict these masked words by the contextual words. (1) To solve the “pre-training and fine-tuning” discrepancy of MLM, MacBERT proposes MLM as correction (Mac) task, which converts the original MLM into a text correction task [71].
2. NSP in BERT is used to determine whether two sentences are contextually related, which is considered not that effective by many studies [61, 72]. MacBERT replaces NSP with the sentence order prediction (SOP) task as introduced by ALBERT [72], which uses two consecutive texts as positive samples, and then switches their original order as negative ones.

Our model uses the MacBERT layer as a component to transform the plant EMR text into vector representations. The textual information is first extracted through three embedding layers: segment embedding, location embedding and word embedding, following which the obtained vector representations are passed to the bidirectional transformer encoder. The core mechanism of Transformer is multi-headed attention, as shown in Eq. 1:

1 $${\text{Attention}}\left( {Q,K,V} \right) = {\text{Softmax}}\left( {\frac{{QK^{T} }}{{\sqrt {d_{k} } }}} \right)V$$

where $Q$, $K$, and $V$ are the input vector matrix, $d_{k}$ represents the dimension of the input vector.

Finally, multiple sets of parameter matrices are introduced for linear transformation and concatenation to obtain the enhanced semantic vector as the output, as shown in Eqs. 2 and 3:

2 $${\text{MultiHead}} (Q,K,V) = {\text{Linear}} ({\text{Concat}} (head_{1} , \cdots ,head_{h} ))$$

3 $$head_{i} = {\text{Attention}} (QW_{i}^{q} ,KW_{i}^{k} ,VW_{i}^{v} ),i = 1,2, \cdots ,h$$

#### Pooling operations

In order to generate a fixed-length representation of variable-length PEMR text, a pooling operation is used to summarize the information contained in the input sequence into a single vector that captures the semantic information of the entire text. Previous studies have verified the influence of pooling operations on the experimental results, indicating the importance of choosing a suitable pooling method for the model being used. In this study, we considered four commonly used pooling strategies in NLP tasks: CLS, mean, First-last avg, and pooler. Each of these methods has distinct functions and differences:

1. CLS: uses the “[CLS]” token directly from the PLM as the vector representation of the entire sentence. CLS pooling is commonly used in tasks that require a simple and efficient approach such as text classification.
2. Mean: calculates the average of each token in the output of the PLM to represent the sentence vector. This approach is simple and computationally efficient, and can capture the overall semantics of the input sequence.
3. First-last avg: uses the average from the combination of the first and last layers of the PLM as the sentence representation. This approach is useful in tasks such as sentiment analysis, where the first and last tokens of a sentence often contain important information about the sentiment.
4. Pooler: puts the “[CLS]” token through a fully connected layer and Tanh activation function as the sentence representation. This approach can be more effective but also requires more computational resources and training data.

#### Loss function

Advanced sentence embedding methods, Sentence-BERT and SimCSE, are considered. Sentence-BERT consists of two parameter-sharing BERT networks, each of which receives a sentence as input and acquires a fixed-length vector of sentence embeddings after a pooling operation. Then the cosine distance of two sentence embeddings is calculated as similarity in the inference stage. But in the training stage, Sentence-BERT uses a classification objective function unrelated to cosine. The classification objective function is described in Eq. 4. Two sentence embeddings $u$ and $v$ are concatenated with the element-wise difference vector $\left| {u - v} \right|$ between them and multiplied by the trainable weight $W_{t} \in R^{3n*k}$.

4 $${\text{s}} = {\text{softmax}} (W_{t} \cdot (u,v,\left| {u - v} \right|))$$

where $n$ is the dimension of the sentence embeddings and $k$ is the value of the target labels. Finally, cross-entropy loss functions are used to train the model.

**Fig. 4:**
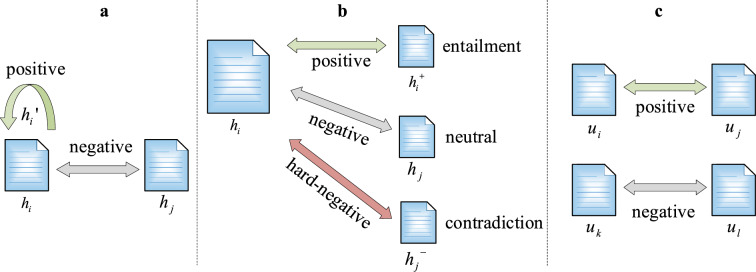
Differences in samples suitable for different models

Without the inconsistent training and prediction objectives of Sentence-BERT, SimCSE optimizes the cosine values directly in training. Unsupervised SimCSE uses sentences with itself as positives and other sentences in the batch as negatives (Fig. 4a). Supervised SimCSE further leverages performance through NLI data labels. The NLI dataset [73] consists of data triads in which the relationship between two sentences is either implicit, neutral or contradictory.

As shown in Fig. 4b, $h_{i}$ and $h_{i}^{ + }$ are positive pairs with labeling entailment while $h_{i}$ and $h_{j}^{ - }$ are hard-negative pairs with labeling contradiction, the training objective is:

5 $$s = - \log \frac{{e^{{\cos (h_{i} ,h_{i}^{ + } )/\tau }} }}{{\sum\nolimits_{j = 1}^{N} {e^{{\cos (h_{i} ,h_{j}^{ + } )/\tau }} + e^{{\cos (h_{i} ,h_{j}^{ - } )/\tau }} } }}$$

where $\tau$ is a temperature hyperparameter.

In contrast to the data triad of the NLI dataset, the format of our target data is the sentence pair (Fig. 4c). To implement the idea of optimizing cosine similarity in SimCSE on our data, the CoSENT loss function proposed by Su (2022) [83] is introduced.

6 $$s = \log (1 + \sum\limits_{{(i,j) \in \Omega_{pos} ,(k,l) \in \Omega_{neg} }} {e^{{\lambda (\cos (u_{k} ,u_{l} ) - \cos ({\varvec{u}}_{{\varvec{i}}} \user2{,u}_{{\varvec{j}}} ))}} } )$$

where $\lambda$ is a hyperparameter.

#### Training process

To ensure reproducibility, we provide a comprehensive description of the training process, including the following steps:

Step 1: Preprocess the PEMR data to ensure that the input data is in a suitable format for training. During training, we set the maximum text length to 80. If the input text exceeds this length, it is truncated, and if it is shorter, it is padded with zeros to match the maximum length.

Step 2: Initialize the PLM Layer and the weights of the pre-trained MacBERT model are loaded, providing a strong initial starting point for training.

Step 3: During training, we employ contrastive learning. By training the model to correctly distinguish between positive and negative pairs, we encourage the model to capture meaningful semantic representations.

Step 4: The AdamW optimizer is used to update the model parameters during training. The learning rate is set to 2e-5, and the batch size is 32. Backpropagation is used to compute the gradients, and the optimizer adjusts the model's parameters accordingly, repeating this process for 15 epochs.

### Hardware, software environment and evaluation metrics

Software Environment: all experimental codes were executed on Python 3.6 with Pytorch1.10.0 and CUDA 11.3. Hardware Platforms: we used a cloud server with RTX 3090(24 GB) CPU, Intel(R) Xeon(R) Gold 6330 CPU and 256 GB memory to train models.

The evaluation of the model is divided into two parts: (1) the ability of the model to estimate the similarity of the electronic medical records. The similarity between two sentence embeddings is evaluated using cosine similarity as the main metric. We calculate Pearson’s Coefficient and Spearman’s Rank Coefficient to indicate how our cosine similarity estimates and the ground truth labels provided by the dataset are related. (2) The accuracy of the model's prescription recommendations in application scenarios. Simulating a real application scenario, plant disease descriptions are used as input to obtain diagnoses and prescriptions based on a standard prescription library. The correctness (Accuracy) of the diagnostic results is counted as an evaluation index of the prescription recommendation effectiveness.

7 $$Accuracy = \frac{{n_{p} }}{{n_{r} }}$$

where $n_{p}$ denotes the number of accurate prescriptions recommended and $n_{r}$ denotes the total number of recommended prescriptions. Considering the diversity and personalization of prescription prescribing in actual agricultural production, the top-n approach is used in this paper for model evaluation.

## Results and discussion

### Semantic matching experimental results

The core of the prescription recommendation model constructed in this study lies in semantic matching, and only a model with excellent semantic matching capability can achieve effective prescription retrieval and recommendation. To validate the semantic matching ability of the proposed model, experiments are conducted in this section on the constructed Vdateset-E, and the model performance is evaluated.

#### Different PLM and different pooling layer

The effects of different PLM structures and pooling types on the model matching effect are compared: four pooling layers are chosen: first-last avg, mean, CLS, max, and three PLMs are chosen: macBERT, BERT, and roBERTa. With 3 kinds PLM models and 4 pooling types, we provide 12 different PLM + pooling results.

The detailed results are shown in Table 1, with the best performance in each column highlighted in bold. The results demonstrate that different PLMs are better suited for different pooling operations. Our model achieved the highest performance by combining MacBERT with CLS pooling, achieving a Pearson coefficient of 86.34% and a Spearman coefficient of 77.67%. MacBERT is trained by masking the word with its similar word rather than using the [MASK] token [71], which helps to improve the understanding of word embeddings [74]. Similarly, RoBERTa is also better suited for CLS pooling. Overall, the CLS pooling method achieved effective results through a simple structure compared to the other three complex pooling methods. This may be because contrastive learning directly updates the representation of [CLS] token [75]. However, BERT performed better when enhanced by mean pooling (MEAN), with a Pearson correlation coefficient of 86.11% and a Spearman correlation coefficient of 77.53%. These results align with the findings of [75], where mean pooling was shown to enhance the semantic correlation between adjacent characters in Chinese when applied to BERT.

**Table 1:** Comparison of different PLMs and different pooling layers

PLM layer	Pooling operations	Pearson (%)	Spearman (%)
BERT	First-last avg	84.84	77.22
RoBERTa	First-last avg	84.6	77.07
MacBERT	First-last avg	85.66	77.10
BERT	Mean	86.11	77.53
RoBERTa	Mean	85.80	77.29
MacBERT	Mean	85.89	77.33
BERT	Pooler	84.40	77.02
RoBERTa	Pooler	84.29	77.11
MacBERT	Pooler	83.83	77.41
BERT	CLS	85.95	77.27
RoBERTa	CLS	85.95	77.16
MacBERT	CLS	86.34	77.67

#### Different sentence embedding solution

**Fig. 5:**
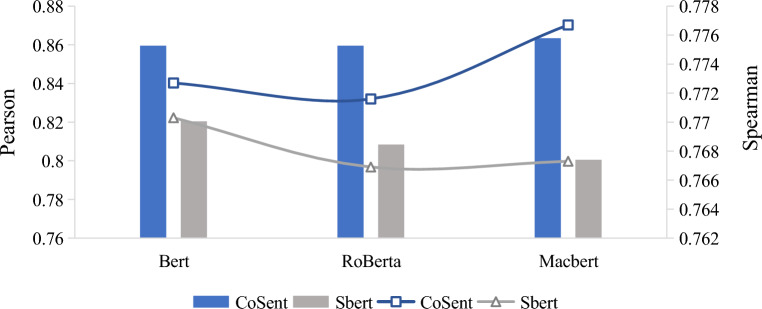
Different Sentence embedding solution

Two sentence embedding schemes are compared: supervised simCSE optimized with the CoSENT loss function (our model) and sentence-BERT (sBERT), both of which employ CLS pooling. Figure 5 displays Pearson's and Spearman's coefficients for the diagnostic results obtained with different combinations of PLM and sentence embedding loss functions. The results indicate that our model outperforms sBERT, irrespective of the PLM used. Notably, the performance gap between CoSENT and sBERT is particularly pronounced when RoBERTa or macBERT is used as PLM layer. Specifically, when combined with RoBERTa, CoSENT attains Pearson's and Spearman's coefficients of 0.8595 and 0.7716, respectively, as opposed to 0.8084 and 0.7669 for sBERT. Similarly, with macBERT, CoSENT achieves Pearson's and Spearman's coefficients of 0.8634 and 0.7767, respectively, while sBERT obtains 0.8005 and 0.7673.

These findings suggest that CoSENT may be better suited for capturing semantic similarity between sentences [79], particularly when more powerful pre-trained models are employed. However, it is noteworthy that all combinations of models yielded Pearson's and Spearman's coefficients above 0.75, indicating that pre-trained language models and sentence embedding schemes are effective for capturing semantic similarity between sentences, corroborating the view of studies such as [66–68].

### Prescription recommendation testing

To verify the prescription recommendation capability of the model in application scenarios, we conduct closed-set tests and open-set tests on the vegetable disease-prescription dataset and the fruit disease-prescription dataset, respectively. The former (Vdateset-Q) is derived from the same distributed space as our model training data (Vdateset-E), while the latter (Fdateset-Q) contains a diverse range of plants and prescription types that the model did not see during training. The experimental results are shown in Table 2, and the bolded font indicates the best results in that column.

**Table 2:** Prescription recommendation testing on closed-set and open-set

Model	Closed-set (%)			Open-set (%)		
	TOP.1	TOP.3	TOP.5	TOP.1	TOP.3	TOP.5
BERT-sBERT	79.67	87.87	91.15	75.24	89.81	92.48
roBERTa-sBERT	79.02	87.21	91.15	76.21	90.53	93.45
macBERT-sBERT	78.69	86.89	90.82	76.94	90.29	95.39
BERT-CoSENT	86.23	96.07	**98.03**	81.07	91.26	94.66
RoBERTa-CoSENT	86.56	95.41	97.05	80.34	**92.72**	93.45
PRSER(our model)	**88.20**	**96.07**	97.70	**82.04**	91.50	**94.90**

The bolded values indicate the best-performing results in each column

#### Closed-set testing

The results show that the method proposed in this paper can achieve good prescription recommendation accuracy in closed-set testing. Specifically, our model achieved Top-1 accuracy of 88.20%, Top-3 accuracy of 96.07%, and Top-5 accuracy of 97.70%. These are good results considering the complexity of the prescription recommendation task that involves multiple vegetables and multiple treatment options.

Although the Top-1 accuracy of BERT-CoSENT is 1.97% lower than that of our model, it achieves the same results as our model in Top-3 and is 0.33% higher than our model in Top-5. In addition, RoBERTa-CoSENT also achieves relatively good results. This indicates that the choice of pre-trained language model has less impact on the accuracy of prescription recommendations.

On the other hand, the sentence embedding scheme has a greater influence on the accuracy of prescription recommendations. The model with CoSENT sentence embedding scheme for prescription recommendation was significantly more effective than the model with SBERT, which is consistent with the results of the previous semantic matching experiments. The CoSENT sentence embedding scheme directly optimizes the cosine values without the problem of inconsistent training and prediction targets of SBERT and has achieved superior results in several NLP tasks [76–78].

#### Open-set verification

Test our prescription recommendation method on the fruit disease dataset to explore its generalization capability. As shown in Table 2, our model achieves good results in the open-set test, although slightly worse than in the closed-set test. Specifically, the Top-1 accuracy of the PRSER model on the open set is 82.04%, which is 6.16% lower than that on the closed set; the Top-3 accuracy is 91.50%, which is 4.57% lower than that on the closed set; and the Top-5 accuracy is 94.90%, which is 2.80% lower than that on the closed set.

The decrease in accuracy compared to the closed-set test is expected, as the training set of the model does not contain fruit-related PEMRs and prescriptions. Nevertheless, the relatively high Top3 and Top5 accuracies indicate that our model can still make good prescription recommendations even for new prescriptions. This is because our prescription recommendation model relies on semantic matching between PEMRs. The training data is significantly extended by transforming disease-prescription pairs into binary-labeled disease-disease pairs. This approach enables the model to concentrate on the semantic extraction of PEMRs and accentuate the distinction between disease information. Consequently, the model is capable of effectively embedding PEMRs for prescription recommendations, even when faced with prescriptions with limited sample sizes or entirely new prescriptions not included in the training data.

### Application scenario analysis

#### Plant disease prescription recommendation system construction

To address the challenge of the limited number of plant doctors and the inability of plant clinics to meet the needs of a large number of farmers, this paper designs and implements a plant disease prescription recommendation system for mobile terminals based on sentence embedding. The system provides farmers with timely and accurate intelligent services, including symptomatic control, scientific use of pesticides, and decision-making support, ultimately improving the quality and efficiency of agricultural production.

The system utilizes Django in conjunction with WeChat applets for mobile development and the Django REST framework to establish a RESTful API for data exchange and communication between mobile devices and the server. In addition, a lightweight SQLite database is employed as a serverless, zero-configuration, transactional SQL database engine for data storage and management.

**Fig. 6:**
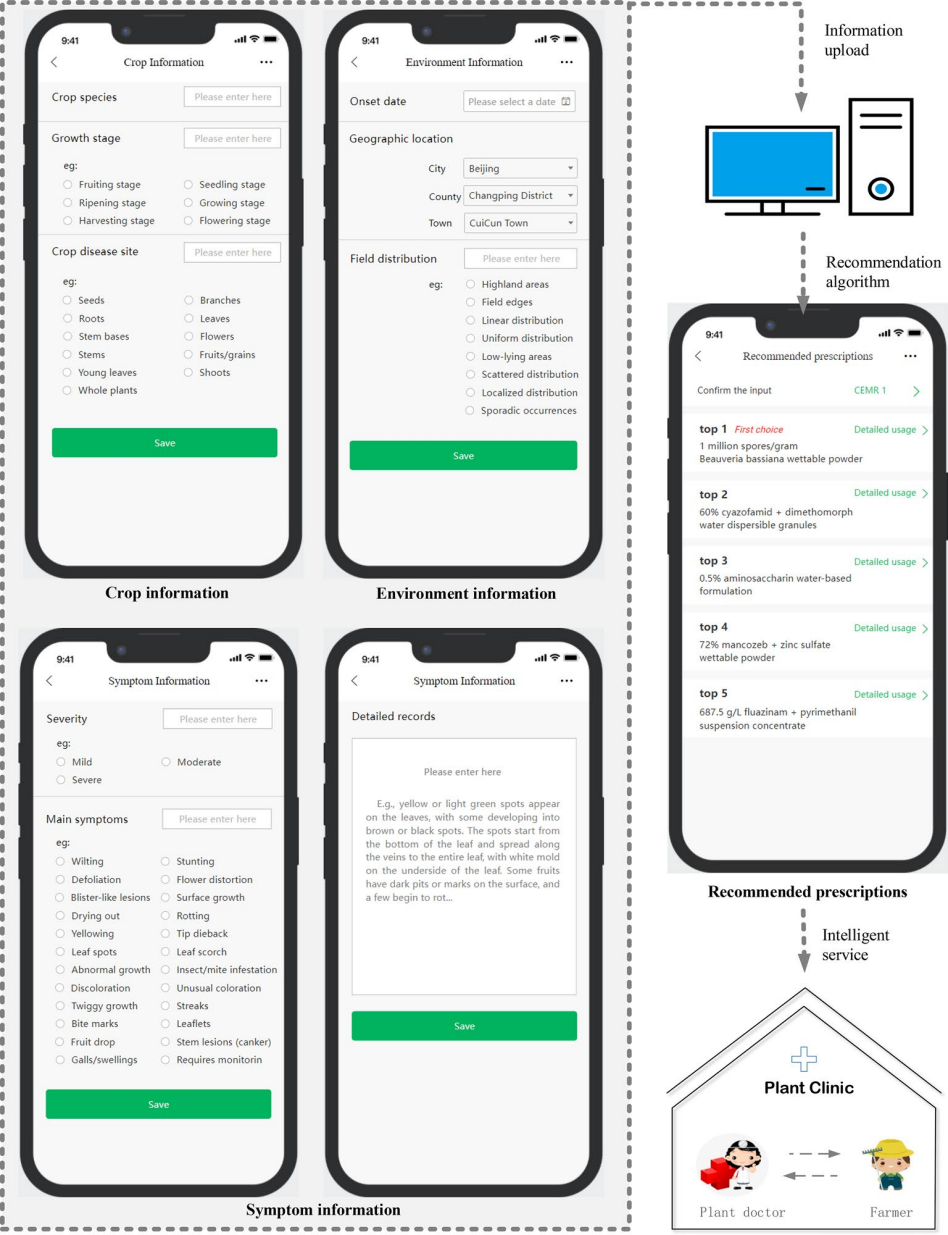
System input–output workflow diagram

Considering the limited experience of system users in describing and recording plant diseases, the system is designed with a detailed mobile app interface to guide users in entering various disease-related information. The system references the widely recognized “disease triangle” principle in plant pathology, which is a triangular framework consisting of pathogen, host and environment [79]. In practical scenarios of diagnosis and treatment of plant diseases, environmental, plant and symptom-related information needs to be considered. To facilitate the input of information, the mobile app interface organizes the information to be entered into three categories: (1) environmental information (onset date, geographic location, field distribution), (2) plant information (plant species, growth stage, and plant disease site), and (3) symptom information (severity, main symptoms, and detailed records). The Mockplus RP software is used to implement the interface display and user interaction, ensuring a user-friendly and intuitive user experience for users. After the user fills in disease-related information, the system generates the top 5 prescription recommendations based on the proposed prescription recommendation model and the constructed prescription reference (as shown in Fig. 6). The system can provide a direct service to farmers as well as a reference for plant doctors.

#### Analysis of common problems in application scenarios

**Fig. 7:**
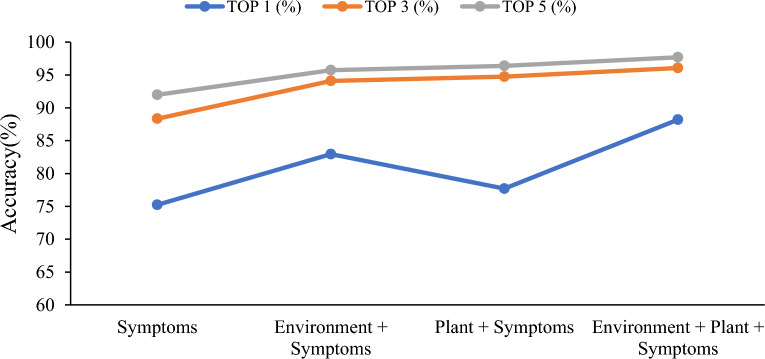
Prescription recommendation result of different inputs on closed-set

**Fig. 8:**
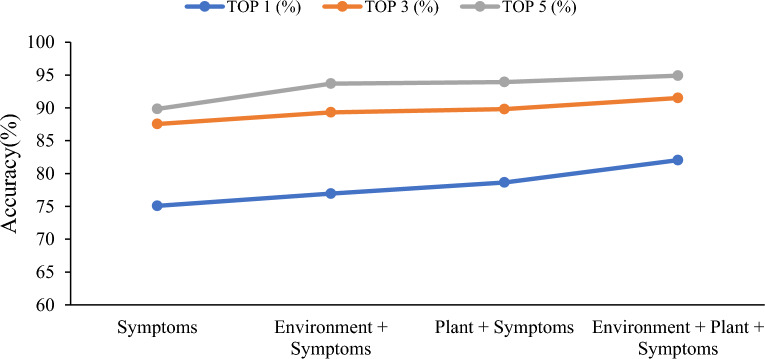
Prescription recommendation result of different inputs on open-set

The previous experiments have demonstrated that the plant disease prescription recommendation system utilizing the PRSER model can produce favorable results for the complete PEMRs filled by plant doctors from plant clinics. However, in practical application scenarios, users may input incomplete data, such as when only symptom information is available without corresponding environmental information. To evaluate the generalization ability of the model in handling incomplete inputs, we conducted experiments using different input methods and evaluated the performance of the model using the TOP1/3/5 accuracy metrics. The experiments were conducted on a dataset with incomplete disease-related information, and the results are shown in Fig. 7 and Fig. 8.

The experimental results demonstrate that the system's recommendation accuracy can be enhanced by improving the completeness of the input data, with symptom information playing a pivotal role. This is attributed to the fact that phenotypic characteristics represent the most intuitive and characteristic manifestations of crop diseases [6, 7]. Specifically, with only symptom information, the system can still provide good accuracy, with top-5 accuracy of 91.99% for the closed-set test and 89.84% for the open-set test. This indicates that the proposed model has good adaptability to incomplete input data.

While the results are promising and demonstrate that incorporating environmental and plant-related information can significantly enhance the accuracy of the recommendation system, it is important to acknowledge certain limitations and potential challenges encountered during the study. The achieved TOP 5 accuracy rate of 97.70% in the closed test and 94.90% in the open test validates the underlying principle of the disease triangle in plant pathology, showcasing the intricate interaction among the environment, plant, and pathogen in disease development [79]. However, we must recognize that these results were obtained under specific conditions and may not be generalizable to all scenarios.

The availability and quality of the input data from users are noteworthy limitations. The accuracy of the recommendations heavily relies on the comprehensiveness and accuracy of the information provided during the practical application of the plant disease prescription recommendation system. If users fail to supply relevant and precise data, it may affect the system's ability to deliver accurate recommendations. For example, with symptom information only, the top-5 accuracy is 91.99% for the closed-set test and 89.84% for the open-set test, which are both lower than the results with full input of Environment + Plant + Symptoms. Therefore, encouraging users to provide comprehensive information is crucial to maximizing the potential of the recommendation system.

## Conclusions and future directions

### Conclusions

The PRSER method proposed in this study demonstrates excellent performance in plant disease prescription recommendations. We constructed a multi-vegetables disease dataset and a multi-fruit disease dataset for comparing different PLMs, pooling operations, and loss functions. The results of the semantic matching experiments show that the combination of MacBERT, CLS pooling, and CoSENT loss function achieves the best performance, with a Pearson coefficient of 86.34% and a Spearman coefficient of 77.67%. Furthermore, the prescription recommendation test results demonstrate that the PRSER exhibits good accuracy in both closed-set and open-set scenarios, with Top-1/Top-3/Top-5 accuracies ranging from 82.04% to 97.70%.

We have further designed and implemented a plant disease prescription recommendation system for mobile terminals. The system features a user-friendly mobile app interface that guides users to enter information related to the environment, plant, and symptoms. Application scenario experiments demonstrate that the completeness of the input data positively affects the recommendation accuracy of the system, with symptom information identified as the most important determinant.

In summary, the PRSER approach proposed in this study has great potential to advance agricultural intelligence by facilitating plant disease prescription recommendations. Our findings provide valuable insights for future research in this area, especially in exploring new data sources, refining the recommendation system, and expanding its applicability in real-world agricultural production.

### Future directions

The PRSER method proposed in this study exhibits outstanding performance in plant disease prescription recommendations. However, there are several avenues for future research and improvement to enhance its capabilities and applicability:

1. Integration of Image Data Modalities: In light of the growing interest in computer vision-based plant disease diagnosis, incorporating image data modalities, such as visible image data or hyperspectral data [6], into our model to construct a prescription recommendation model based on multimodal fusion represents a promising research direction.
2. Integration of Other Relevant Data Sources: Exploring the integration of diverse data sources, including weather data, soil characteristics, and historical disease records, could significantly enrich the recommendation system's understanding and decision-making process.
3. Improvement of the Plant Disease Prescription Recommendation System: Two key aspects warrant attention. Firstly, enhancing the interpretability of the prescription system [80], allowing users to comprehend the reasoning behind the AI recommendations [81], would increase user understanding and acceptance. Secondly, refining the system's interaction design to foster seamless human and intelligent system interactions, such as employing question and answer systems, chatbots, and other agents [82], will elevate the overall user experience.

## Data Availability

The datasets used and/or analysed during the current study are available from the corresponding author on reasonable request.

## Footnotes

1 Plantwise is a global, CABI-managed program, aiming at strengthening plant health systems through plant clinics: www.plantwise.org
